# Meeting Report from “Frontiers in Nutritional Science: Nutritional Metabolomics”

**DOI:** 10.3390/nu6093451

**Published:** 2014-09-03

**Authors:** Nathan O’Callaghan, Manny Noakes

**Affiliations:** CSIRO, Food and Nutrition Flagship, PO Box 10041, Adelaide, SA 5000, Australia

## 1. Preface

The potential for transforming nutritional and health research through the discovery and application of non-invasive markers of dietary intake and metabolic status is profound. The science of metabolomics for the fingerprinting of volatile organic compounds (VOCs) from expired human breath holds great promise in this regard. Coupled with tools utilising sensor technology, breath volatile signatures allow a new horizon of research in which indicators of metabolic risk and indicators of dietary intake could be collected at a population level with unprecedented simplicity and low cost. Metabolomics (measuring metabolites from physiological process) provides a “window into the body”, which could transform how we measure health, how we identify and monitor people most at risk of disease and the way we monitor food intake.

To stimulate interest and cooperation in this frontier field of health sciences, in August 2013, CSIRO in collaboration with the University of South Australia, and ILSI Southeast Asian Region organised a symposium which aimed to explore opportunities for using metabolomics to improve human health by nutritional means. This one-day symposium brought together international and national experts to provide a comprehensive, contemporary overview of this emerging field. The event aimed to provide food and health science professionals and researchers, an exciting trajectory of how nutritional metabolomics may be applied to enlarge our understanding of how food, diet and the body interact. In particular, this symposium connected researchers from diverse fields of metabolomics. These ranged from physicians and researchers applying breath tests for disease diagnosis, sensory scientists with a knowledge of volatile compounds in foods, nutritional physiologists exploring breath compounds following dietary interventions to engineers developing sensor technologies for point of care testing. This rich diversity of disciplines fostered a day of many exciting discussions and potential collaborations.

We were fortunate to have three international experts present their work to a highly engaged audience: Prof Augustin Scalbert (IARC) presented on using the food metabolome to explore interactions between diet and health/disease in epidemiological studies; Prof Ong Choon Nam (NUS) spoke emphatically on utilising metabolomics as part of biomarker discovery platform; and Prof Fredrick-Jan van Schooten (Maastricht University) presented his exciting work on breath volatiles in clinical science to monitor health. These international experts were supported by a range of National leaders (Manny Noakes, Páraic O’Cuív, Ute Roessner, Stephen Trowell, Conor Delahunty, Peter Meikle, Ross Butler, Nathan O’Callaghan and Graeme Woodrow); abstracts from the presentations are included here to enable this knowledge to be shared with the nutritional science community.

Following the successful completion of the symposium, the speakers as well as key delegates were invited to participate in a workshop the following day. The workshop aimed to foster collaborations, identify what is state of the art in metabolomics, what are the potential applications and what are the gaps (data analysis, tools, *etc*.). Prof Graeme Woodrow acted as a facilitator and emphatically challenged the workshop participants with some key questions: How will metabolomics transform health and well being in the future? What are the key research directions and questions? How do we get there?

There was broad ranging consensus that these technologies have the potential as a non invasive platform to measures a person’s health status as well as to enable to characterise what people eat. However, to realise the potential of nutritional metabolomics, there is a need to develop a collaborative network to build a research agenda for the necessary research and data that will bring the vision to fruition. In this regard, this inaugural symposium marks one step forward in linking researchers in this field.

## 2. Summary of Scientific Presentations

### 2.1. Opportunities for Metabolomics in Nutritional Science

#### Manny Noakes

Amongst the greatest challenges in human nutrition is characterising dietary intakes in the short and long term and relating these exposures to health outcomes. To date, self reported dietary behaviours have profound limitations, both in terms of diet quantity (energy intake) and quality (nutrient intake). Whilst there are limited objective biomarkers of some dietary exposures, the list is too sparse and too invasive for routine use in nutrition research. The potential for transforming nutritional and health research through the discovery and application of non-invasive markers of dietary intake and metabolic status is profound. In particular, the use of metabolomics for the fingerprinting of volatile organic compounds (VOCs) from expired human breath, coupled with tools utilising sensor technology, allow a new horizon of research in which indicators of metabolic risk and indicators of dietary intake could be collected at a population level with unprecedented simplicity and low cost. In the future, VOCs or saliva, urine, fingerprick blood could enable non invasive approaches to monitoring health using biosensors also as measurement tools for clinicians and consumers, not only researchers. Perhaps through the application of metabolomics in human nutrition through non-invasive methodologies we will ultimately realise the aspiration of personalised nutrition which has yet to be realised.

### 2.2. Metabolomics, a Promising Approach to Explore Interactions between Diet and Health and Diseases in Epidemiological Studies

#### Augustin Scalbert

Major discoveries on the role of essential nutrients in the human organism have been made in the last century using classical hypothesis-driven approaches. However, foods are more than a sum of essential nutrients. They are highly complex mixtures of natural compounds, additives and contaminants, and many of these compounds show some biological activities. Understanding the contribution of the diet to the risk of chronic diseases such as diabetes, cardiovascular diseases or cancers constitutes a considerable challenge for researchers of the 21st century. Reductionist approaches largely failed to identify key factors and mechanisms underlying the complex interactions between diet and health. Modern analytical techniques now allow to measure hundreds when not thousands metabolites as part of the metabolome, the most downstream biochemical readout of individual phenotypes. The systematic comparison of metabolic profiles of individuals exposed to different diets or to varying risk of diseases using metabolomics approaches, opens exciting avenues to better understand the role of the diet in the aetiology of chronic diseases. This will be illustrated with examples of applications in nutritional and cancer epidemiology. Main challenges met in the application of metabolomics to cohort studies will also be emphasized.

### 2.3. How to Utilise Metabolomics and Metagenomics in Nutritional Science

#### Páraic O’Cuív

The microbial community (microbiota) resident in the human gut provides a variety of physiological and ecological functions relevant to host health and well-being. The vast majority of gut microbes are unrepresented by cultured isolates and much of our current understanding of the functional capacity of the gut microbiota has been facilitated by culture-independent metagenomic approaches. These approaches have revealed that the gut microbiota is comprised of a “metagenome” as much as 150 times greater than that of the human genome, and that may be considered to be an extension of the host genotype itself. Much remains to be discovered about the Genotype × Environment × Lifestyle interactions that underpin microbial colonisation and persistence in the gut but it is now known that nutritional factors play a key role in modulating the colonisation potential and functional activity of the gut microbiota. The microbiota encodes a plethora of bioactive metabolites and peptides that impact the host physiological response and whose production is affected by nutritional factors; however, many of the genetic factors underpinning these functionalities remain cryptic. Our current efforts within the Australian Human Gut Microbiome Project have focused on bridging the gap between microbial metagenomics and metabolomics and elucidating the relationship between nutrition, the microbiome and host health.

### 2.4. How do We Measure Metabolomics Profiles?

#### Ute Roessner

In the last two decades, significant progress has been made in the sequencing of the genomes of a number of different organisms. Simultaneously, large investments have occurred in the development of high throughput analytical approaches to analyse different cell products, such as those from gene expression (transcriptomics) as well as proteins (proteomics) and metabolites (metabolomics). These ‘omics approaches, when used in combination with sophisticated bioinformatic tools for data mining and interpretation, are considered important tools to be applied and utilised to understand the biology of an organism and its response to environmental stimuli or genetic perturbation. The ability to measure metabolites in any given biological system and most importantly to determine the relationship of those to the genome, transcriptome and proteome will enhance our ability to better understand biological systems and the consequences of perturbation. In this paper, I want to introduce the current state-of-the-art technologies in the metabolomics sciences. This is then related to available capabilities currently offered through Metabolomics Australia, a national service facility providing access to the analytical and computational requirements for metabolomics from small (simple experiment) to large scale (clinical) studies. 

### 2.5. Developing Sensors to Measuring Breath VOCs for Health and Disease

#### Stephen Trowell

Expired breath and other volatile organic compounds (VOCs) evolved by the organism provide a non-invasive window into metabolism. Potentially, they can be used to diagnose diseases or track metabolic status. This can be done using state of the art analytical equipment such as GC-MS or GC-GC-MS but these are very expensive systems that can only be operated by experts in a laboratory setting. We need an alternative, if point of care VOC analysis is to become useful. Over 30 years ago, Persaud and Dodd invented the concept of the “Electronic Nose” (e-nose), an array of chemical sensors modelled on the human nose. In principle, e-noses are ideal for VOC-based diagnosis but, in practice, the sensor arrays in such devices lack sufficient discriminating power (Berna *et al.* 2009) to reliably detect the signal, often present at parts per billion, against a complex and varying background. We will describe development of a new type of biophotonic sensor and its incorporation into a sensing system, the CYBERNOSE^®^, which has many of the key features required for robust point of care VOC analysis.

### 2.6. Utilising Breath to Understand Nutrition

#### Conor Delahunty

The process of nutrition involves the assimilation of foods for energy, growth, and repair of our bodies. The metabolic processes involved in nutrition include anabolism (the build up of substances) and catabolism (the breakdown of substances) in tissues, leading to an ever changing metabolite composition based upon physiology, nutritional status and metabolic health. Breath analysis is akin to a “headspace analysis” of blood via gas exchange in the lungs, enabling quantification of volatile metabolites present at ppb concentrations. Where blood includes metabolite composition representative of tissues, breath analysis can enable interrogation of nutritional status, and using dynamic measures, of metabolic control. Real-time breath analysis post-consumption of a food can be used as a measure of host × food metabolic interaction. The real-time identification and quantification of nutritional metabolites as they increase and decrease in concentration will advance knowledge of nutritional status in relation to metabolic health by diagnosing individual differences, specific effects of food properties, and likely mechanisms. We have conducted a pilot study, applying Proton Transfer Reaction-Mass Spectrometry (PTR-MS) to measure breath metabolites post-consumption of a food. This study quantified individual dynamic “fingerprints” of volatile organic compounds in subjects, which were indicative of nutritional status. 

### 2.7. Applications for Lipidomics in Clinical Nutrition

#### Peter Meikle

A growing body of population based studies have demonstrated an association between diet and risk of type 2 diabetes and cardiovascular disease. However, the effects are often inconsistent and confounded by the complexities of assessing specific foods in a complex dietary environment. Such studies often require large numbers to achieve adequate power to identify significant associations. Metabolic disorders that increase risk of type 2 diabetes and cardiovascular disease are often characterized by dyslipidemia, leading to inflammatory and oxidative processes. Lipidomics enables a detailed assessment of dyslipidemia beyond the traditional measures of cholesterol, lipoproteins and triglycerides and so can provide additional insight to the metabolic processes associated with chronic disease and potentially the effects of diet on these processes. We have performed lipidomic analysis of participants from the AusDiab study to characterise the plasma lipid profile associated with prediabeties and type 2 diabetes. Multiple lipid classes including phospholipids, sphingolipids, glycerolipids and sterols were examined. Logistic regression analyses identified associations with type 2 diabetes (135 lipids) and prediabetes (134 lipids), after adjusting for multiple covariates. In addition to the expected associations with diacylglycerol, triacylglycerol and cholesterol esters, type 2 diabetes and prediabetes were positively associated with ceramide, and its precursor dihydroceramide, along with phosphatidylethanolamine, phosphatidylglycerol and phosphatidylinositol. Significant negative associations were observed with the ether-linked phospholipids, alkylphosphatidylcholine and alkenylphosphatidylcholine, and some lysophospholipids. In subsequent studies we, and others, have identified similar associations of some of same lipid species with high fat diets, while the opposite associations were observed with dairy foods, thereby providing putative links between diet and chronic disease for future investigations. Source of funding: this research was funded by the Dairy Health and Nutrition Consortium (Australia), The National Institutes of Health (USA) and the National Health and Medical Research Council (Australia). 

### 2.8. Exhaled VOCs in Clinical Science for Monitoring Human Health

#### Frederick van Schooten

In ancient times, physicians valued human breath as a window of diseased and healthy organs. For instance, advanced liver disease was indicated by the fishy smell of a patient’s breath. Thousands of volatile organic compounds (VOCs) are produced in different organs which are transported by blood to the lungs where they are released. Inflammatory and deviant metabolic processes change the composition of these compounds which can be of use for clinical diagnosis and disease monitoring. Nowadays breath analysis makes use of the latest technological innovations such as sophisticated analytical instrumentations and learning algorithm softwares. We have recently developed a Gas Chromatography-Mass Spectrometry-based platform to measure all VOCs (volatome) in exhaled breath. The approach is combined with chemometric analysis that allows extracting the maximal information from the wealth of data obtained. In our approach, we first apply a variable selection procedure to pick up the significant VOCs and next use them to construct a final classification model such as Random Forests (RF). Our statistical approach is validated twice, *i.e.*, within the RF algorithm using an “out-of-bag” prediction error estimate and by using a completely independent validation set. In this presentation, case studies are presented using our technology platform to identify unique compounds associated with inflammatory related conditions concerning the pulmonary tract, intestinal tract and liver. As a first example we have previously shown in several studies that VOCs can be indicative of malfunctions occurring in the lungs as for instance in smokers and groups of patients with Chronic Obstructive Pulmonary Disease (COPD). COPD is an inflammatory condition and we found that oxidative stress related VOCs are sufficient to predict correctly 92% of patients from healthy controls. A second example is to demonstrate the feasibility of exhaled VOCs to monitor different status of human’s gut health, in our case monitoring disease severity in Inflammatory Bowel Disease (IBD). For that purpose we have accrued 313 IBD patients into a prospective, diagnostic study of IBD. For Crohn’s diseased patients, 238 samples from active and 497 from inactive (remission) forms of the disease were established during hospital visits. We found a VOCs profile of a limited set of compounds predicting disease severity with 85% of correct classification. A second illustration of monitoring the gut is a dietary intervention study in which healthy volunteers were following a gluten free diet for four weeks. Each week breath samples and stool were collected. The main objective was to investigate what the influence of diet is on exhaled breath and gut microbiome. A number of exhaled VOCs changed clearly because of the dietary intervention and in further research this will be related to a changed in microbiome. The last example is on expanding the experience of ancient physicians that diseased livers smell. In this study we analyzed exhaled breath of obese patients undergoing surgery. Some obese patients develop non-alcoholic steatohepatitis (NASH) which is associated with liver failure and mortality. Our aim was to address this unmet medical need by searching for diagnostic VOCs related to NASH in obese patients. In a prospective study 65 overweight and obese subjects, undergoing either routine laparoscopic cholecystectomy or laparoscopic bariatric surgery, were included. Breath was sampled pre-operatively, and wedge liver biopsies were obtained during surgery and histologically evaluated according to the NASH activity. It was observed that three exhaled compounds were sufficient to distinguish subjects with (*n* = 39) and without NASH (*n* = 26), with an area under the ROC curve of 0.77. This breath test showed negative and positive predictive values of 82% and 81%, indicating that in more than 80% of subjects, the prediction of both absence or presence of NASH was correct. This is relatively high compared to assessment based upon current radiological techniques or risk assessment as performed by plasma transaminase levels. Breath analysis as non-invasive method holds promise to be of importance in monitoring purposes and clinical practice. Non-invasive monitoring technologies have the potential to revolutionize modern medicine moving from a reactive treatment mode into a preventive and personalized mode facilitated by point-of-care and home-based diagnostic tools.

### 2.9. Utility of Breath to Measure Disease

#### Ross Butler

Breath tests have been used as accepted clinical tools more widely in gastroenterological disease diagnosis and management than in possibly any other speciality. Isotopically labelled compounds (usually 14C and 13C) were first adopted in the early 1970s with specialty Units carrying them out clinically. The 13C urea breath test for detection of H.pylori infection, now the preferred non-invasive method for diagnosis and for monitoring efficacy of antibiotic therapy, has been the most successful and robust isotopic breath test developed. Additionally a range of gut function tests including 13C breath tests for lipase activity for pancreatic insufficiency, and gastric emptying for motility disorders have been adopted in some geographic regions. Breath tests for disaccharide malabsorption (sucrose and lactose) and more recently the monosaccharide fructose malabsorption have been in use for several decades. These breath tests use the H2 production of the large bowel flora as the marker of carbohydrate malabsorption. All of these breath tests need the oral administration of a probe substrate. In most jurisdictions only the *H. pylori* Breath Test and the Breath H2 Test for carbohydrate malabsorption are reimbursable on Medical Benefits Schedules. With the gut microbiome assuming centre stage in human health, the understanding and defining of fermentative and disease signatures of volatile organic compounds (VOCs), in addition to CH4 and H2 expired in breath, now assumes more importance. Mechanistic links and new management paradigms for both intestinal disease (IBS, IBD) and extraintestinal disorders, e.g., obesity, metabolic syndrome, NASH and cancers, will follow.

### 2.10. Breath Volatiles to Monitor Metabolic Health

#### Nathan O’Callaghan

It has been recognised for millennia that expired breath volatiles, the trace chemicals present in the exhaled breath of humans, represent a snapshot of the metabolic state of the individual. In classical times, clinicians used their sense of smell to diagnose diseases. The modern era of breath analysis was initiated by Linus Pauling, who applied a GC-MS to human breath and demonstrated the presence of more than 200 distinct volatile chemicals. Recent advances in metabolomics of human breath have found that it can be used as a non-invasive diagnostic of health status, including ageing, inflammation and oxidative stress, lung diseases, and metabolic disorders. In this regard, it was recently reported that breath analysis can identify individual metabolic phenotypes, demonstrating application in personalised health. Breath analysis of key volatiles enables us to interrogate anabolic conditions (after food intake) and catabolic states (fasting) can be an expressed as a marker of the plasticity of metabolic control. These metabolic processes can be monitored through our ability to measure specific compounds, as indicators of an individual’s metabolic health. Recent metabolomics studies have indicated that plasma free fatty acids, acylcarnitines, ketone bodies, branched chain amino acids, and amino acid degradation products are significantly altered in insulin resistance or diabetes. Interestingly, insulin-resistant or diabetic patients show metabolite profiles of a catabolic condition, even in an otherwise anabolic state. Here we report on preliminary results investigating dynamics of breath volatiles during a weight loss intervention in an obese cohort with type 2 diabetes. Breath samples were captured over a 12-week period during a calorie restricted dietary intervention. Breath VOC we measured by GC-MS. We aim to explore whether certain breath VOC are associated with weight loss and/or glycaemic control.

### 2.11. Breath VOCs for Health and Disease Diagnostics: A Point-of-Care Diagnostic Platform Based on Inexpensive Sensors and Mobile Phones

#### Graeme Woodrow

There is still a major need for diagnostic platforms that meet the stringent cost and performance criteria required for deployment in remote and resource poor areas. Most tests based on immunochemical or DNA detection of pathogens fail to meet these criteria. We are taking a metabolomic approach to the diagnosis of infectious disease in the developing world in which metabolite patterns of either the pathogen or the host characteristic of a particular disease are identified. In a collaboration involving the Nossal Institute for Global Health at the University of Melbourne, the CSIRO and supported by the Science and Industry Endowment Fund (SIEF), we bring together three components in order to achieve this, viz. chemiresistor-based sensors, algorithms running on a simple microcontroller and mobile phones for final analysis and data presentation. Overall, the combination will be simple to use, inexpensive and have high performance. The other clear advantage is that detection does not rely on a blood sample, but can utilize samples obtained from non-invasive sources such as breath, saliva or urine. This technology clearly also has applications as a point- of-care diagnostic platform for the developed world.

## 3. Affiliations

- Butler, R., Senior Cancer Research Fellow Division of Health Sciences Sansom Institute for Health Research University of South Australia Biography
- Delahunty, C., Food and Nutrition, CSIRO
- Meikle, P., Head, Metabolimics Laboratory and Metabolomic (Lipidomic) Profiling Facility Baker IDI Heart and Diabetes Institute
- Noakes, M., Research Director, Food and Health, CSIRO
- O’Callaghan, N., Food and Nutrition Flagship, CSIRO
- O’Cuív, P., Research Scientist, Food and Nutrition, CSIRO
- Roessner, U., Head of Analytics, Metabolomics Australia Node leader, Australian Centre for Plant Functional Genomics, School of Botany, The University of Melbourne
- Scalbert, A., Group Head, Nutrition and Metabolism - Biomarkers Group International Agency for Research on Cancer World Health Agency, Lyon, France
- Trowell, S., Theme Leader, Quality Biosensors CSIRO Ecosystem Sciences
- van Schooten, F., Department of Toxicology, Faculty of Health, Medicine and Life Sciences, Research Institute NUTRIM, Maastricht University, The Netherlands
- Woodrow, G., Health Strategy and Implementation CSIRO

